# Phase 1b randomized, double-blind study of namilumab, an anti-granulocyte macrophage colony-stimulating factor monoclonal antibody, in mild-to-moderate rheumatoid arthritis

**DOI:** 10.1186/s13075-017-1267-3

**Published:** 2017-03-09

**Authors:** T. W. J. Huizinga, A. Batalov, R. Stoilov, E. Lloyd, T. Wagner, D. Saurigny, B. Souberbielle, E. Esfandiari

**Affiliations:** 1 Leiden University Medical Center, Albinusdreef 2, PO Box 9600, 2300RC Leiden, The Netherlands; 2 Medical University of Plovdiv, UMHAT Kaspela, Plovdiv, Bulgaria; 3 University Hospital (MHAT) St Ivan Rilski, Sofia, Bulgaria; 4 Takeda Pharmaceuticals International, Deerfield, IL USA; 5 Takeda Pharmaceuticals International GmbH, Zurich, Switzerland; 6 Takeda Pharmaceuticals International, 61 Aldwych, London, WC2B 4AE UK

**Keywords:** GM-CSF, Namilumab, Phase 1b, Rheumatoid arthritis

## Abstract

**Background:**

Namilumab (AMG203) is an immunoglobulin G1 monoclonal antibody that binds with high affinity to the GM-CSF ligand. This was a phase 1b, randomized, double-blind study (PRIORA) to assess namilumab in active, mild-to-moderate rheumatoid arthritis (RA). The primary outcome was the safety and tolerability of repeated subcutaneous injections of namilumab in patients with mild-to-moderate RA.

**Methods:**

Adults with mild-to-moderate RA on stable methotrexate doses for ≥12 weeks were eligible. Patients received three subcutaneous injections of namilumab 150 or 300 mg, or placebo on days 1, 15, and 29, with 12 weeks’ follow-up. Primary objective was safety/tolerability.

**Results:**

Patients in cohort 1 were randomized to namilumab 150 mg (*n* = 8) or placebo (*n* = 5). In cohort 2, patients were randomized to namilumab 300 mg (*n* = 7) or placebo (*n* = 4). Incidence of treatment-emergent adverse events (TEAEs) was similar across the three groups (namilumab 150 mg: 63%; namilumab 300 mg: 57%; placebo: 56%). TEAEs in ≥10% of patients were nasopharyngitis (17%) and exacerbation/worsening of RA (13%). No anti-namilumab antibodies were detected. The pharmacokinetics of namilumab were linear and typical of a monoclonal antibody with subcutaneous administration. In a post hoc efficacy, per protocol analysis (*n* = 21), patients randomized to namilumab showed greater improvement in Disease Activity Score 28 (erythrocyte sedimentation rate and C-reactive protein [CRP]), swelling joint counts and tender joint counts compared with placebo. Difference in mean DAS28-CRP changes from baseline between namilumab and placebo favored namilumab at both doses and at all time points. In addition area under the curve for DAS28-CRP was analyzed as time-adjusted mean change from baseline. A significant improvement in DAS28-CRP was shown with namilumab (150 and 300 mg groups combined) compared with placebo at day 43 (*p* = 0.0117) and also 8 weeks after last dosing at day 99 (*p* = 0.0154).

**Conclusions:**

Subcutaneous namilumab was generally well tolerated. Although namilumab demonstrated preliminary evidence of efficacy, patient numbers were small; phase 2 studies are ongoing.

**Trial registration:**

ClinicalTrials.gov, NCT01317797. Registered 18 February 2011.

**Electronic supplementary material:**

The online version of this article (doi:10.1186/s13075-017-1267-3) contains supplementary material, which is available to authorized users.

## Background

Rheumatoid arthritis (RA) is a complex, chronic, autoimmune disease characterized by joint inflammation leading to erosions of articular cartilage and subchondral bone [1, 2]. Despite advances in treatment with biologic disease-modifying antirheumatic drugs (DMARDs), a significant proportion of RA patients are still not adequately controlled. For example, most patients treated with biologic DMARDs do not achieve 50% or 70% improvement according to the American College of Rheumatology criteria (ACR50 or ACR70 responses). Only a small proportion of patients achieve remission with biologic DMARDs, and responses are often not durable, necessitating frequent treatment switching [3, 4]. This lack of adequate disease control indicates a need for new therapies with innovative mechanisms of action for those patients who fail to achieve remission or low disease activity, developing resistance to treatment response, or experience significant toxicities with current therapy.

Granulocyte macrophage colony-stimulating factor (GM-CSF) is a hematopoietic growth factor produced by a number of different cell types, including: T cells, macrophages, mast cells, endothelial cells, smooth muscle cells, epithelial cells, and fibroblasts [5–8]. GM-CSF stimulates the proliferation and activation of mature myeloid cells inducing the production of inflammatory molecules, thereby acting as a pro-inflammatory cytokine [6]. As GM-CSF is a key activator of the innate immune system, it is likely to play an important role in the pathogenesis of autoimmune inflammatory diseases (including RA) in which macrophages, neutrophils, granulocytes, eosinophils, and dendritic cells contribute to disease progression [5, 9, 10]. In patients with RA, GM-CSF is aberrantly overproduced [11–15]; GM-CSF levels are moderately elevated in the plasma and highly elevated in the synovial fluid [14, 16], particularly in the pannus at sites of cartilage erosion [17]. The contribution of GM-CSF to the development of RA has also been documented in various in vitro and in vivo mouse models [18–23]. Moreover, exacerbation of RA disease activity has been reported in patients receiving GM-CSF as supportive therapy to resolve neutropenia in Felty’s syndrome or post-chemotherapy [24, 25].

The central role of GM-CSF in immune responses and its involvement in autoimmune inflammatory diseases supports the rationale for GM-CSF-targeted therapy as a novel treatment approach for RA. Proof-of-concept for GM-CSF-targeted therapy has been demonstrated for antibodies targeting the GM-CSF receptor and soluble GM-CSF [26–31].

Namilumab (AMG203) is a human immunoglobulin G1 (IgG1) monoclonal antibody that binds with high affinity to the GM-CSF ligand, potently neutralizing GM-CSF [32]. Preclinical data showed that a surrogate mouse antibody of namilumab (22E9) neutralized GM-CSF, suppressed inflammation, and protected cartilage in an arthritis mouse model [33]. In a first-in-human study, healthy volunteers showed that namilumab (single doses up to 8.0 mg/kg) were generally well tolerated [34].

This phase 1b, first-in-patient, multicenter, randomized, double-blind, placebo-controlled, dose-escalation study assessed the safety, tolerability, pharmacokinetic (PK) and pharmacodynamic (PD) characteristics and preliminary efficacy of repeated subcutaneous injections of namilumab in patients with active mild-to-moderate RA on stable doses of methotrexate.

## Methods

### Patients

Patients aged ≥18 years, diagnosed with active RA (according to ACR 1987 revised criteria), on stable doses of methotrexate (≥7.5– ≤ 25 mg/week) for at least 12 weeks before the first dose of study drug and with low-to-moderate disease activity (Disease Activity Score 28 [DAS28]-erythrocyte sedimentation rate [ESR] ≥2.6– ≤ 5.1) were included. Concomitant nonsteroidal anti-inflammatory drugs (NSAIDs) with appropriate gastro-protection, low-dose corticosteroids (≤10 mg prednisone equivalence per day) and hydroxychloroquine (≤400 mg/day) were permitted at stable doses for at least 4 weeks prior to the first dose of study drug.

Exclusion criteria included: unstable RA disease status with flares; significant extra-articular manifestations of RA (e.g., pulmonary fibrosis or vasculitis); use of other DMARDs except methotrexate; and presence or history of major chronic inflammatory autoimmune diseases (such as psoriasis, inflammatory bowel disease or systemic lupus erythematosus). Patients with a medical history of methotrexate-associated lung toxicity, a history of severe pulmonary disease, and a clinically relevant decrease in lung function (forced expiratory volume in 1 second [FEV_1_] <70% of the predicted value) were also excluded from the study since GM-CSF inhibition could affect alveolar macrophage function and surfactant homeostasis in the lung (see Discussion section) [34, 35]. Previous use of GM-CSF and/or granulocyte colony-stimulating factor and concomitant medication (except methotrexate, NSAIDs, low-dose corticosteroids, and hydroxychloroquine) was not permitted.

### Study design

Patients received a single subcutaneous injection of namilumab or placebo on days 1, 15, and 29, in addition to continued treatment with methotrexate. Two dose levels of namilumab were evaluated: 150 mg (cohort 1) and 300 mg (cohort 2). Patients were followed for approximately 12 weeks after the last dose of study drug (Additional file 1: Figure S1).

Institutional review boards/ethics committees at the participating investigational centers approved the study, which was conducted according to the principles set out in the Declaration of Helsinki, International Conference on Harmonisation Guidelines for Good Clinical Practice, and additional local regulations.

### Objectives and assessments

The primary objective was to evaluate the safety and tolerability of repeated subcutaneous injections of namilumab in patients with mild-to-moderate RA. Secondary objectives included evaluation of PK and PD (namilumab/GM-CSF complexes), including explorative biomarker assessments and immunogenicity. Clinical efficacy was an exploratory objective. A predefined, post hoc efficacy analysis was also performed on the PRIORA data to evaluate the clinical effects of namilumab on the signs and symptoms of RA (see Statistical analyses section).

Safety was assessed by adverse events (AEs); clinical laboratory parameters, urinalysis, electrocardiogram (ECG), vital signs, pulmonary function tests (FEV_1_, forced vital capacity [FVC] and peak flow at specified time points); and physical examination. Anti-namilumab antibodies were quantified using a bridging electrochemiluminescence assay. PD assessments included analysis of namilumab/GM-CSF complexes, as well as assessment of peripheral blood cytokines and other markers of inflammation.

In the initial analysis, the exploratory objective of efficacy was assessed using ACR20 and DAS44-ESR. In the post hoc efficacy analysis, DAS28-C-reactive protein (DAS28-CRP), DAS28-ESR, tender joint count (TJC; 68 joints), swollen joint count (SJC; 66 joints) and patient’s global disease activity (100 mm visual analog scale [VAS]) were evaluated in a per protocol population. In order to minimize the bias in the post hoc efficacy analysis, the statistical analysis plan and the criteria for the per protocol population were specified prior to the analysis. CRP was quantified at a central laboratory, while ESR was measured locally.

### Statistical analyses

Safety, PD and efficacy populations included all patients who received namilumab or placebo; and for whom safety, tolerability or efficacy data were available. The PK population included all patients who received at least one dose of namilumab and for whom PK parameters could be calculated.

The post hoc analyses were conducted on the per protocol population and excluded any patients with major protocol criteria violations which could potentially affect clinical efficacy (e.g., those who had used prohibited concomitant medications, or those who were not on a stable background dose of methotrexate or corticosteroids). Patients who increased their background corticosteroid dose during the study, or used any DMARDs other than methotrexate, were classified as non-responders and, where applicable, any subsequent data after the treatment violation were excluded from the post hoc analyses.

Statistical analyses, which were primarily descriptive, were undertaken using a statistical software program (SAS system, version 9.2, SAS Institute, Inc., Cary NC, USA). Namilumab plasma concentration data were analyzed in WinNonlin (Phoenix® WinNonlin® version 6.3, Certara Inc., St. Louis, MO, USA) by non-compartmental analysis using a plasma model with extravascular dose type and the actual sampling time. DAS28 (CRP and ESR) data are expressed as mean (standard deviation) changes from baseline. Differences between namilumab (both doses combined) and placebo in the mean change from baseline in DAS28-CRP were determined, along with 95% confidence intervals (CI), at each visit. The rationale for this analysis was to assume that both doses would perform better than placebo and that the comparison with placebo of the two doses combined would negate any potential cohort effect. An analysis of the DAS28-CRP profile (area under the curve; AUC) was performed by determining the time-adjusted mean change from baseline; comparisons to placebo were tested using a Wilcoxon test. Improvements in DAS28-ESR were defined according to European League Against Rheumatism (EULAR) response criteria [36].

## Results

### Patients

A total of 24 patients were enrolled at 10 sites (2 in The Netherlands, 4 in Bulgaria, and 4 in Spain). The first patient was randomized March 2011, the last patient was enrolled April 2013, and the last patient visit was August 2013. Patient demographics and baseline characteristics are shown in Table 1. Thirteen patients were included in cohort 1 (8 randomized to namilumab 150 mg and 5 to placebo) and 11 were included in cohort 2 (7 randomized to namilumab 300 mg and 4 to placebo). The median age was 59 years (range 29–75), most patients were female (71%), and almost all patients were white (96%). Mean baseline DAS28-ESR was 4.7; disease activity was moderate and similar across treatment groups. A total of 22 patients completed the study (2 patients discontinued: 1 patient randomized to placebo in cohort 1 [due to patient withdrawal] and 1 patient randomized to namilumab 300 mg in cohort 2 [due to the patient leaving the country]; (Additional file 2: Figure S2). Protocol amendments did not impact data or study outcome.

**Table 1:** Baseline patient demographics and disease characteristics (safety population)

	Placebo (n = 9)	Namilumab 150 mg (n = 8)	Namilumab 300 mg (n = 7)	Total (N = 24)
Gender, n (%)				
Female	6 (67)	5 (63)	6 (86)	17 (71)
Male	3 (33)	3 (38)	1 (14)	7 (29)
Race, n (%)				
White	9 (100)	7 (88)	7 (100)	23 (96)
Black	0	1 (13)	0	1 (4)
Age, years^a^	56 (29–65)	59 (43–65)	59 (36–75)	59 (29–75)
BMI, kg/m^2b^	27.37 (2.246)	24.69 (2.471)	28.30 (1.778)	26.75 (2.607)
Disease duration, years	3.3 (0.9–10.9)	4.9 (1.7–19.0)	5.2 (3.0–9.3)	4.4 (0.9–19.0)
DAS28-ESR^b^	4.8 (0.41)	4.9 (0.35)	4.4 (0.59)	4.7 (0.48)
DAS28-CRP^b^	4.4 (0.82)	4.2 (0.52)	4.0 (0.62)	4.2 (0.65)
ESR, mm/hour^b^	31 (11.91)	28 (5.56)	23 (8.06)	28 (9.31)
CRP, mg/liter^b^	21 (22.64)	8 (6.75)	12 (10.44)	13 (15.43)
TJC^b^ (0–68)	8.7 (5.45)	9.1 (6.92)	9.6 (4.69)	9.1 (5.55)
SJC^b^ (0–66)	5.0 (5.41)	4.3 (2.38)	6.1 (5.30)	5.1 (4.45)

The eligibility criteria allowed patients with previous biological therapy into the study, however, all of patients enrolled into the PRIORA study were biologic naive

*BMI* body mass index, *CRP* C-reactive protein, *ESR* erythrocyte sedimentation rate, *SD* standard deviation, *SJC* swollen joint count, *TJC* tender joint count

^a^Median (range); ^b^mean (SD)

### Safety

A total of 49 treatment-emergent AEs (TEAEs) were reported in 14 patients (58%) (Table 2). The percentage of patients who had any TEAE was similar between the treatment groups (placebo: 56%; namilumab 150 mg: 63%; namilumab 300 mg: 57%). The most frequent TEAEs were nasopharyngitis (*n* = 4; 17%), exacerbation or worsening of RA (*n* = 3; 13%), musculoskeletal pain (*n* = 2; 8%), and increased blood creatine phosphokinase (*n* = 2; 8%). Two serious TEAEs were reported in the namilumab 150 mg group. One was in a 61-year-old female smoker who received three doses of namilumab and was diagnosed with severe non-small cell lung cancer 14 months after the last dose; this was the only reported severe TEAE. The other serious TEAE was in a 61-year old male with a history of coronary artery stenosis and abnormal ECG, who received and tolerated three doses of namilumab, and was diagnosed with mild coronary artery stenosis following a routine ECG test on the day of the second dose. Both serious TEAEs were considered unrelated to namilumab. Five TEAEs were considered to be treatment related: 3 in the placebo group (increased blood creatine phosphokinase, bradycardia, and somnolence) and 1 in each namilumab group (150 mg: increased alanine aminotransferase; 300 mg: increased blood creatine phosphokinase). These were considered non-serious and did not lead to study discontinuation or changes in study medication. There were no deaths reported during this study.

**Table 2:** TEAEs in >1 patient by system organ class

System organ class, n^a^ (%)	Placebo (n = 9)	Namilumab 150 mg (n = 8)	Namilumab 300 mg (n = 7)	Total (N = 24)
Preferred term, n^a^ (%)				
Any TEAE	5 (56)	5 (63)	4 (57)	14 (58)
Musculoskeletal and connective tissue disorders	3 (33)	3 (38)	0	6 (25)
Exacerbation/worsening of RA	2 (22)	1 (13)	0	3 (13)
Musculoskeletal pain	0	2 (25)	0	2 (8)
Pain in extremity	1 (11)	0	0	1 (4)
Muscular weakness	1 (11)	0	0	1 (4)
Laboratory investigations (total)^b^	1 (11)	3 (38)	2 (29)	6 (25)
Infections and infestations	2 (22)	3 (38)	0	5 (21)
Nasopharyngitis	2 (22)	2 (25)	0	4 (17)
Urinary tract infection	0	1 (13)	0	1 (4)
Gastrointestinal disorders	0	2 (25)	1 (14)	3 (13)
Abdominal pain, upper	0	1 (13)	0	1 (4)
Diarrhea	0	1 (13)	0	1 (4)
Abdominal pain	0	0	1 (14)	1 (4)
Cardiac disorders	1 (11)	1 (13)	0	2 (8)
Bradycardia	1 (11)	0	0	1 (4)
Coronary artery stenosis	0	1 (13)	0	1 (4)
General disorders and administrative site conditions	1 (11)	0	1 (14)	2 (8)
Chest discomfort	1 (11)	0	0	1 (4)
Chest pain	0	0	1 (14)	1 (4)
Influenza-like illness	0	0	1 (14)	1 (4)
Nervous system disorders	1 (11)	1 (13)	0	2 (8)
Paresthesia	0	1 (13)	0	1 (4)
Somnolence	1 (11)	0	0	1 (4)
Renal and urinary disorders	1 (11)	0	1 (14)	2 (8)
Dysuria	1 (11)	0	0	1 (4)
Nephrolithiasis	0	0	1 (14)	1 (4)

*RA* rheumatoid arthritis, *TEAE* treatment-emergent adverse event

^a^Number of patients with ≥1 event in the category; ^b^of which: increased blood creatine phosphokinase (n = 2; 8%)

### PK

Namilumab plasma concentrations following three consecutive single subcutaneous injections of namilumab (150 or 300 mg), administered 2 weeks apart, were quantifiable for 84 days (last PK sampling time point). The PK-evaluable population included all 8 patients in the namilumab 150 mg group and 7 patients in the namilumab 300 mg group.

**Fig. 1:**
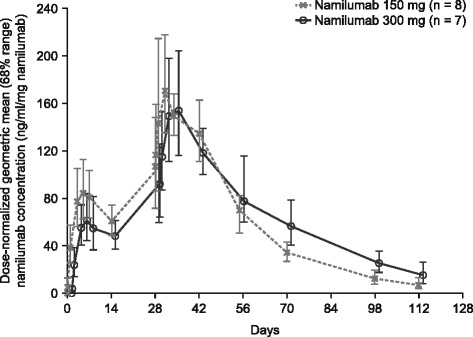
Dose-normalized geometric mean plasma concentration–time profile of namilumab (error bars show ± 1 SD). *SD* standard deviation

The dose-normalized geometric mean plasma concentration–time profiles are shown in Fig. 1. The PKs of namilumab were linear and typical of an IgG1 monoclonal antibody administered subcutaneously. The maximum observed plasma concentration (C_max_) was reached at 5 to 6 days (T_max_) after the first and third injection. Mean terminal half-life (t_1/2_) values were approximately 3 weeks. The dose-normalized exposure was similar for both groups. Anti-namilumab antibodies were not detected in any patient.

### PD

GM-CSF/namilumab complexes increased over time reaching its maximum on day 43 for the 150 mg group and on day 56 for 300 mg group, respectively. At the end of the trial, levels were still above baseline for both groups.

There were no significant or consistent changes in peripheral blood cytokines or pro-inflammatory markers, including: interleukin-1 (IL-1), IL-6, IL-8, monocyte chemotactic protein-1 (MCP-1), tumor necrosis factor alpha (TNF-α), vascular endothelial growth factor (VEGF) or matrix metalloproteinase 3 (MMP-3), related to namilumab administration (data not shown).

### Clinical efficacy

Efficacy was an exploratory objective using DAS44-ESR and ACR20 assessment. In an initial analysis, mean and median DAS44-ESR showed a general decrease from baseline in all treatment groups including placebo. On days 27 and 43 (2 weeks after the last namilumab dose), the 300 mg namilumab group had the most pronounced decrease (mean DAS44 reduction: 0.995 and 0.852, respectively) compared with the placebo group (mean DAS44 reduction: 0.383 and 0.469, respectively). Mean DAS44 reduction from baseline in the 150 mg namilumab group was 0.798 on day 27 and 0.873 on day 43. From day 56 (4 weeks after the last namilumab dose), mean DAS44 reduction from baseline started decreasing in the 150 mg namilumab group; however, in contrast, there was a more pronounced response in the placebo group. This pronounced response in the placebo group was influenced by 2 patients. One in particular had severe disease activity up to day 43 (DAS44 5.24 at day 43), and showed a fast response (DAS44 decreased to 1.43 at day 56) after receiving high-dose methylprednisolone, sulfasalazine, and hydroxychloroquine in addition to methotrexate. Mean DAS44 reduction from baseline increased in the 300 mg namilumab group until day 56 and, thereafter, remained almost unchanged until day 99.

The initial analysis also demonstrated that in all treatment groups, including placebo, and at all visits from day 13, there were patients who met the ACR20 criteria. Although ACR20 was higher numerically in the 300 mg namilumab group compared with the placebo group at all visits, the results were inconclusive in terms of a clear efficacy signal because of a high ACR20 response in the placebo group, especially after day 43.

The post hoc analysis assessed DAS28 in a per protocol population in order to undertake an additional investigation of the clinical significant effects of namilumab on the signs and symptoms of RA using the DAS28, SJC (66 joints), TJC (68 joints), and patient outcome measures (VAS scores). These analyses were conducted on all subjects in PRIORA and on a predefined subset of patients who were free from major protocol criteria violations, which could potentially affect clinical efficacy. Three patients were excluded: 1 patient in the namilumab 150 mg group and 1 patient in the placebo group due to changes in dose of corticosteroids and/or methotrexate prior to randomization; and 1 patient in the placebo group due to receiving a high dose of corticosteroid (intramuscular methylprednisolone 120 mg) and an additional DMARD (sulfasalazine) during the study, as well as changes in dose of corticosteroids prior to randomization. Baseline patient demographics and disease characteristics of the per protocol population are shown in Table 3.

**Table 3:** Baseline disease characteristics of the per protocol population

Mean (SD)	Placebo (n = 7)	Namilumab 150 mg (n = 7)	Namilumab 300 mg (n = 7)	Total (N = 21)
DAS28-ESR	4.7 (0.33)	4.9 (0.35)	4.4 (0.59)	4.7 (0.47)
DAS28-CRP	4.0 (0.35)	4.4 (0.33)	4.0 (0.62)	4.1 (0.46)
ESR, mm/hour	31.3 (7.43)	27.7 (5.94)	23.0 (8.06)	27.3 (7.66)
CRP, mg/liter	16.4 (25.09)	9.1 (6.42)	11.5 (10.44)	12.2 (14.91)
TJC (0–68)	6.6 (2.15)	10.0 (6.98)	9.6 (4.69)	8.7 (5.00)
SJC (0–66)	3.4 (1.4)	4.7 (2.14)	6.1 (5.3)	4.8 (3.42)
PGA for pain	54.6 (14.9)	58.4 (21.1)	50.1 (16.7)	54.3 (17.5)
PGA of DAS	42.4 (21.1)	59.9 (19.3)	43.3 (16.4)	39.5 (18.9)

*CRP* C-reactive protein, *DAS* disease activity score, *ESR* erythrocyte sedimentation rate, *PGA* patient global assessment, *SD* standard deviation, *SJC* swollen joint count, *TJC* tender joint count

**Fig. 2:**
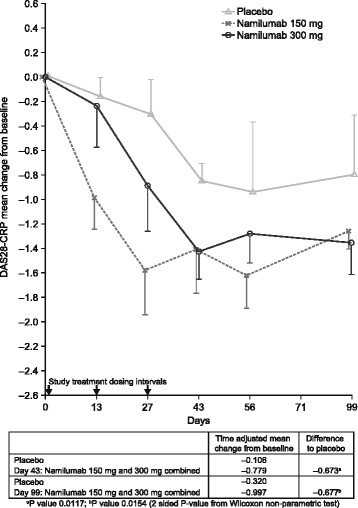
Change from baseline in DAS28-CRP with namilumab compared with placebo. ^*^Error bars show upper SE for placebo and lower SE for namilumab. *DAS* disease activity score, *CRP* C-reactive protein, *SE* standard error

**Fig. 3:**
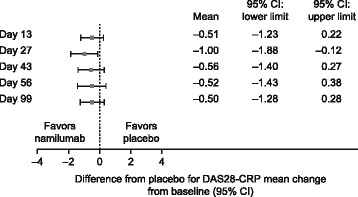
Forest plot showing the difference from placebo with namilumab for DAS28-CRP mean change from baseline. *CI* confidence interval, *CRP* C-reactive protein, *DAS* disease activity score

Two weeks after the last dose (day 43), reductions in DAS28 (ESR and CRP) and joint counts were greater with namilumab (150 and 300 mg) compared with placebo. Namilumab (150 and 300 mg), compared with placebo, was associated with greater improvements in DAS28-CRP as early as day 27; these improvements were maintained until the end of the study (day 99; Fig. 2) specifically for the 300 mg namilumab cohort. A significantly greater improvement in DAS28-CRP was shown with namilumab (150 and 300 mg groups combined) compared with placebo at day 43 (−0.779 vs −0.106, *p* = 0.0117) and also day 99 (−0.997 vs −0.320, *p* = 0.0154), as measured by the time-adjusted mean change from baseline (per protocol subset), (Fig. 2). Individually, the time-adjusted mean change of DAS28-CRP from baseline for namilumab 150 mg and namilumab 300 mg at day 43 was −0.940 vs −0.106 (*p* = 0.0140) and −0.620 vs −0.106 (*p* = 0.0734), respectively, and at day 99 was −1.165 vs −0.320 (*p* = 0.0140) and −0.829 vs −0.320 (*p* = 0.1014), respectively. The differences in mean DAS28-CRP changes from baseline between namilumab (150 and 300 mg groups combined) and placebo favored namilumab at all time points (Fig. 3). DAS28-ESR followed a similar pattern, although the data were more variable. On day 56 (4 weeks after the last dose), a greater proportion of patients had a DAS28-ESR response (>1.2 decrease from baseline) with namilumab (71% [*n* = 10/14]; both groups combined) compared with placebo (29% [*n* = 2/7]). The DAS28-ESR response rate was 86% (*n* = 6/7) in the namilumab 150 mg group and 57% (*n* = 4/7) in the namilumab 300 mg group. SJCs and TJCs showed a greater decrease over time in patients who received namilumab compared with placebo. The observed improvement in SJCs and TJCs with namilumab versus placebo was apparent from the first dose and maintained at all subsequent time points (Additional file 3: Figure S3). Improvements in patient-reported outcome measures, including patient global assessment of disease activity and assessment of pain were also higher in the namilumab-treated groups than the placebo group.

## Discussion

This phase 1b study assessed the safety, tolerability, PK, PD, and preliminary efficacy of repeated subcutaneous injections of namilumab in patients with mild-to-moderate RA on stable doses of methotrexate. Namilumab subcutaneous injections (150 and 300 mg) given every 2 weeks for 4 weeks were generally well tolerated with an acceptable safety profile. Incidence of TEAEs was similar between treatment groups and reported AEs were mostly mild in intensity and similar between the namilumab and placebo cohorts. The majority of TEAEs with moderate intensity were reported in patients receiving placebo. The only TEAE of severe intensity was a non-small cell lung cancer that occurred in a 61-year-old female smoker who was diagnosed 14 months after the last dose of namilumab (150 mg); furthermore, it was not considered to be related to the study medication. No drug reactions, allergic reactions or injection-site reactions were reported during the trial. The safety profile observed for namilumab in our study was consistent with previously reported clinical experience using anti-GM-CSF antibodies. In addition, in accordance with data from a preclinical, multiple-dose toxicology study in monkeys [34], there was no indication of immunogenicity to repeated subcutaneous administration of namilumab in this study, as indicated by the lack of anti-namilumab antibodies in blood samples taken from patients.

Autoantibodies against GM-CSF have been associated with the development of idiopathic autoimmune pulmonary alveolar proteinosis (PAP), a rare but potentially serious lung disease, in which abnormal accumulation of pulmonary surfactant protein occurs within the alveoli due to insufficient clearance by GM-CSF-starved macrophages [37, 38]. Because of this association, there is a theoretical risk of developing PAP when using therapeutic antibodies that target GM-CSF. Accordingly, patients with severe pulmonary diseases were excluded from the study and pulmonary function tests were employed in this study as part of a safety monitoring plan, even though the risk of developing PAP after short-term repeated administration of namilumab was considered very low. Importantly, no abnormal respiratory signals were detected in our study. A recently reported, long-term, phase 2a, open-label extension study also demonstrated an acceptable safety profile with no significant pulmonary signals when mavrilimumab was administered over 74 weeks to patients with moderate-to-severe RA on a stable dose of methotrexate [39].

In the PK/PD analyses, namilumab plasma concentrations following three consecutive single subcutaneous injections of 150 or 300 mg showed typical absorption and elimination kinetics associated with a human IgG1 antibody administered via this route [40]. The PK of namilumab was linear with a T_max_ of 5–6 days and a t_1/2_ of approximately 3 weeks. The PD analyses showed an increase in GM-CSF/namilumab complexes after namilumab injections, which was consistent with the proposed mode of action for namilumab [32]. Further PD analyses showed no changes in peripheral blood cytokines or other markers of inflammation following namilumab administration. A similar lack of effect on pro-inflammatory cytokines was reported for the anti-GM-CSF antibody, MOR103 [30]. In contrast, mavrilimumab has been shown to induce dose-dependent decreases in biomarkers (such as IL-6, MMP-3, serum amyloid A [SAA] and YKL-40) when measured with a multi-biomarker disease activity (MBDA, Vectra DA) score [27], but MBDA was not assessed in the PRIORA study. These apparently conflicting results, as compared with the present study, could be a consequence of the differing sensitivities of the assays used or may reflect differences in systemic inflammation between the two patient populations. Alternatively, it may be that GM-CSF blockade with namilumab does not induce changes in peripheral markers of inflammation. Larger studies are required to further investigate the effect of namilumab on biomarkers of disease activity.

The clinical efficacy analyses were exploratory and were assessed initially using DAS44-ESR from all patients. Although a more prominent reduction in DAS44-ESR from baseline was observed in namilumab-treated patients compared with placebo, particularly in those who received the 300 mg dose, no meaningful or definitive efficacy conclusions could be drawn from the initial analysis. The lack of a marked treatment effect on DAS44-ESR, particularly during the post-dose follow-up period, may have been a consequence of changes in the background RA treatment regimen and/or the effect of decreasing acute phase proteins in the placebo group (baseline CRP was most pronounced in this group and therefore may have contributed to the high placebo response). At the same time, the effects of active treatment were not sustained in the two namilumab-treated cohorts. Nonetheless, a post hoc, per protocol efficacy analysis revealed a strong efficacy signal for namilumab on the signs and symptoms of RA based on its effect on DAS28-CRP, SJCs, and TJCs. This analysis, which was based on a statistical analysis plan incorporating additional clinically relevant endpoints, included 21 patients without major protocol violations (1 patient on namilumab 150 mg and 2 patients on placebo were excluded). The analysis showed that patients receiving namilumab (150 and 300 mg) had greater improvements from baseline in DAS28-CRP, TJCs, and SJCs compared with patients who received placebo. These improved efficacy outcomes with namilumab were reported as early as week 2, as has been reported for mavrilimumab [26–29]. Considering the size of our study, it was an appropriate approach to analyze the DAS28-CRP profile (AUC) in the 150 and 300 mg namilumab combined group versus placebo. A significant improvement in DAS28-CRP was shown with the namilumab combined group compared with placebo at day 43 (*p* = 0.0117) and also 8 weeks after last dosing at day 99 (*p* = 0.0154); these findings provide further support demonstrating the signal efficacy of namilumab in RA patients. Individual treatment comparisons to placebo are more difficult to interpret due to the small subject numbers and possible cohort effect; however the 150 mg namilumab group was statistically different to placebo (*p* = 0.0140) and not the 300 mg group (*p* = 0.1014). Furthermore, the effect of namilumab on DAS28-CRP was more prolonged in those patients who responded to the 300 mg dose compared to those in the 150 mg group, likely reflecting the greater and longer systemic PK exposure at the higher dose.

It was clear from the post hoc efficacy analysis that not all namilumab-treated patients responded well to treatment. Exploratory analyses showed that the lack of effect of namilumab in these poorly responding patients could not be explained by differences in baseline characteristics or PK exposure compared with patients who exhibited moderate or strong responses (data not shown). Exploratory studies are ongoing aimed at identifying biomarkers that can be used to identify a target patient population who will respond to namilumab.

## Conclusions

The results of this study suggest that namilumab (150 and 300 mg) is generally well tolerated and shows signs of clinical activity in patients with mild-to-moderate RA. There are some limitations of this study; it was an early phase, short-duration study with a small number of patients in each treatment group, with some differences in baseline characteristics between groups. Despite these limitations, this study showed a strong signal of efficacy and supports the further development of namilumab for the treatment of RA. A phase 2 dose-finding study of namilumab in combination with methotrexate (NEXUS; NCT02379091) is now ongoing in patients with moderate-to-severe RA who have responded inadequately to methotrexate or an anti-tumor necrosis factor inhibitor.

## Additional files

Additional file 1: Figure S1. PRIORA study design. *D* day (PDF 7 kb)
Additional file 2: Figure S2. Flow diagram showing patient disposition. *DMARDs* disease-modifying antirheumatic drugs, *HIV* human immunodeficiency virus, *IgG*, immunoglobulin G (PDF 16 kb)
Additional file 3: Figure S3. Change from baseline in swollen (a) and tender (b) joint counts. ^*^Error bars show upper SE for placebo and lower SE for namilumab. *SE* standard error, *SJC* swollen joint count, *TJC* tender joint count. (PDF 1292 kb)

## Abbreviations

ACR: American College of Rheumatology criteria
AEs: Adverse events
AUC: Area under the curve
BMI: Body mass index
CI: Confidence interval
C_max_: Maximum observed plasma concentration
CRP: C-reactive protein
DAS: Disease activity score
DMARDs: Disease-modifying antirheumatic drugs
ECG: Electrocardiogram
ESR: Erythrocyte sedimentation rate
EULAR: European League Against Rheumatism
FEV_1_: Forced expiratory volume in 1 second
FVC: Forced vital capacity
GM-CSF: Granulocyte macrophage colony-stimulating factor
IgG1: Immunoglobulin G1
IL: Interleukin
MBDA: Multi-biomarker disease activity
MCP-1: Monocyte chemotactic protein-1
MMP-3: Matrix metalloproteinase 3
NSAIDs: Nonsteroidal anti-inflammatory drugs
PAP: Pulmonary alveolar proteinosis
PD: Pharmacodynamic
PK: Pharmacokinetic
RA: Rheumatoid arthritis
SD: Standard deviation
SJC: Swollen joint count
t_1/2_: Terminal half-life
TEAEs: Treatment-emergent adverse events
TJC: Tender joint count
T_max_: Time to C_max_
TNF-α: Tumor necrosis factor alpha
VAS: Visual analog scale
VEGF: Vascular endothelial growth factor
