# Supplementary figures and images for: Phase 1b randomized, double-blind study of namilumab, an anti-granulocyte macrophage colony-stimulating factor monoclonal antibody, in mild-to-moderate rheumatoid arthritis
Source: Arthritis Res Ther. 2017 Mar 9;19:53. doi: 10.1186/s13075-017-1267-3 (PMC5343373; doi:10.1186/s13075-017-1267-3)

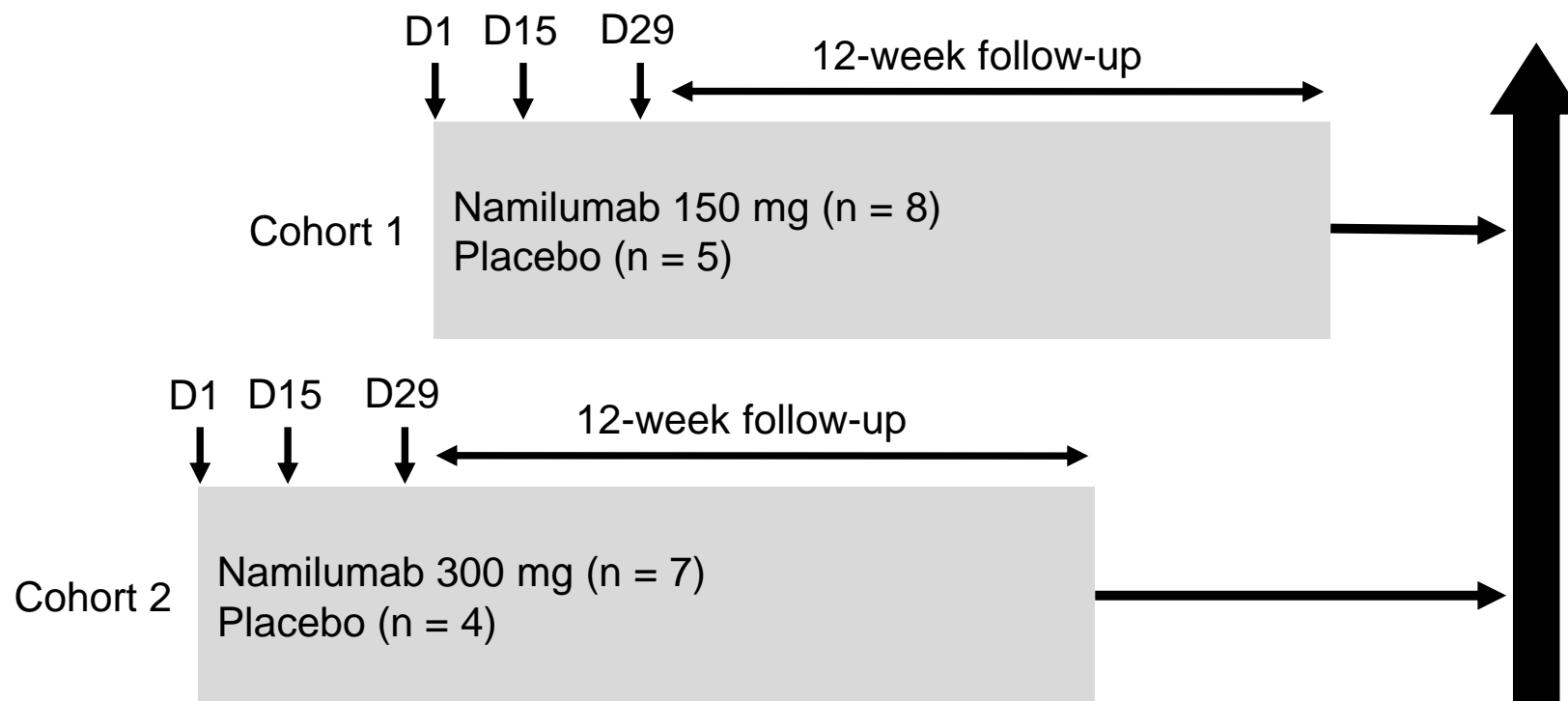

Supplement: Additional file 1: Figure S1. — PRIORA study design. D day (PDF 7 kb) [file 13075_2017_1267_MOESM1_ESM.pdf]

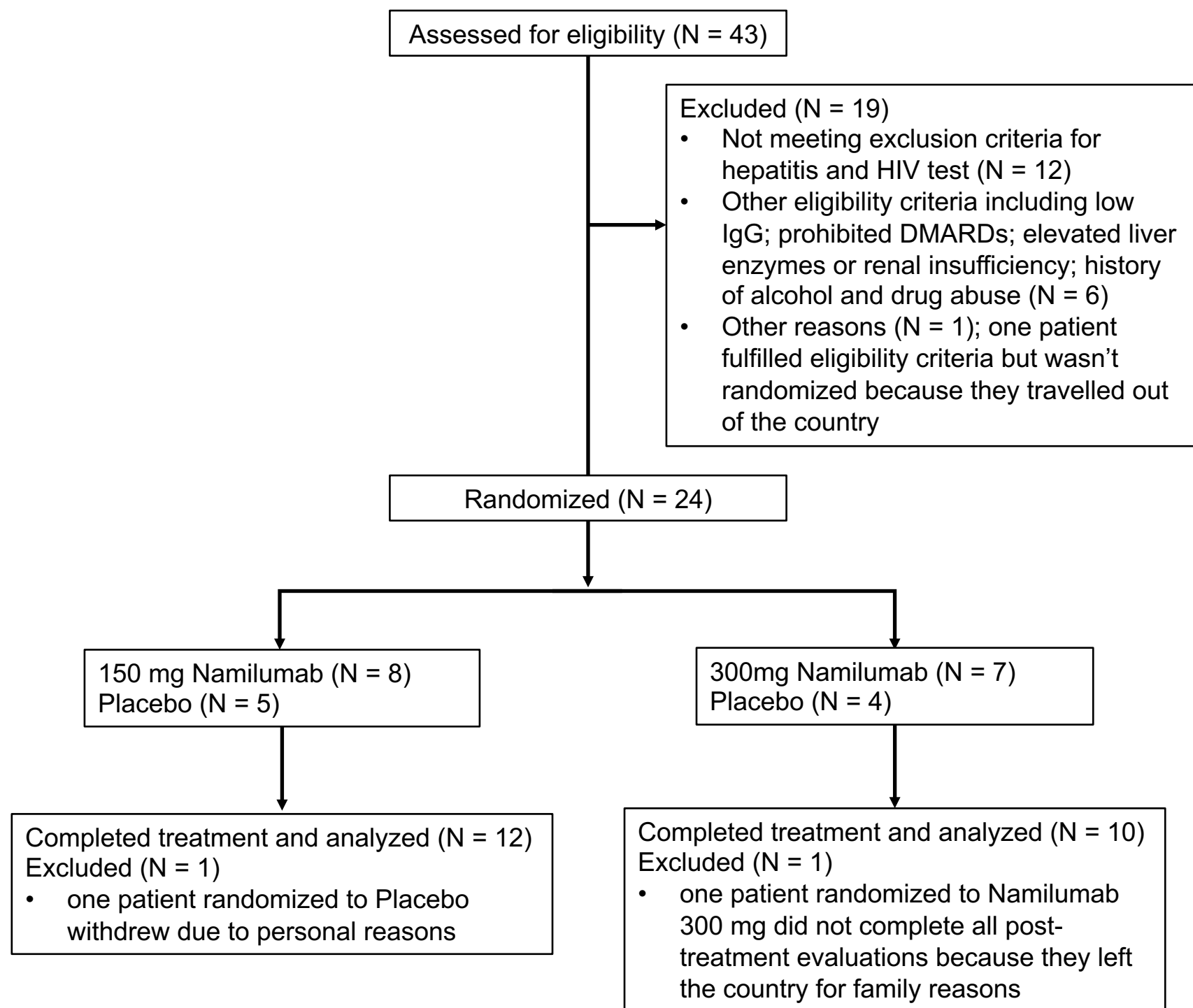

Supplement: Additional file 2: Figure S2. — Flow diagram showing patient disposition. DMARDs disease-modifying antirheumatic drugs, HIV human immunodeficiency virus, IgG, immunoglobulin G (PDF 16 kb) [file 13075_2017_1267_MOESM2_ESM.pdf]

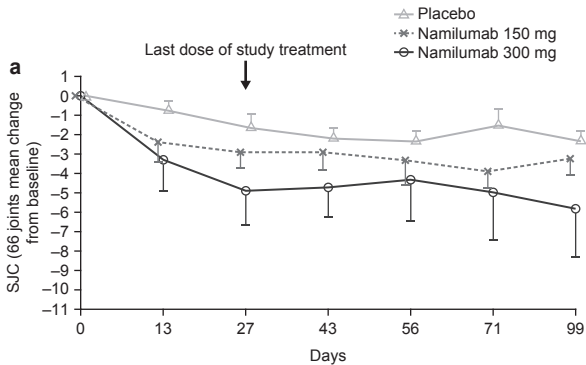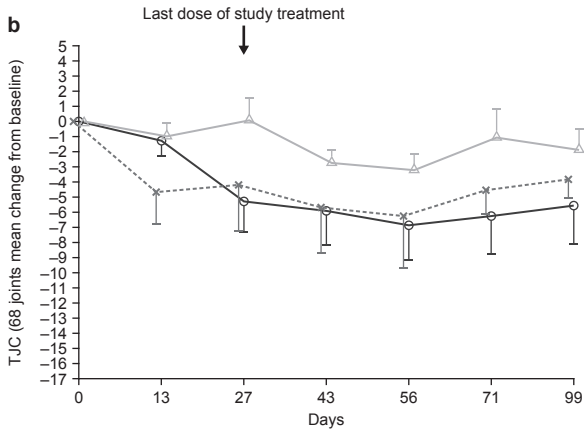

Supplement: Additional file 3: Figure S3. — Change from baseline in swollen (a) and tender (b) joint counts. *Error bars show upper SE for placebo and lower SE for namilumab. SE standard error, SJC swollen joint count, TJC tender joint count. (PDF 1292 kb) [file 13075_2017_1267_MOESM3_ESM.pdf]
